# Lamb Wave-Based FDM-PPM Method Data Transmission Scheme in Plate Structures

**DOI:** 10.3390/s25061907

**Published:** 2025-03-19

**Authors:** Tong Xu, Bin Wu, Xiang Gao, Jianfeng Liu, Xiucheng Liu

**Affiliations:** 1 College of Mechanical & Energy Engineering, Beijing University of Technology, Beijing 100124, China; xutong2020@emails.bjut.edu.cn; 2 School of Information Science and Technology, Beijing University of Technology, Beijing 100124, China; wb@bjut.edu.cn (B.W.); xiuchliu@bjut.edu.cn (X.L.); 3 Taian Special Equipment Inspection and Research Institute, Taian 271000, China

**Keywords:** lamb wave, frequency-division multiplexing, pulse-position modulation

## Abstract

Lamb wave-based non-electromagnetic communication is an effective solution for real-time information exchange in health monitoring networks of large metallic plate structures. The multimodal nature, dispersive characteristics, and the influence of reflected waves during the propagation of Lamb waves severely limit the duration of communication signals. Within this constrained time, constructing communication signals reasonably is crucial for improving the transmission rate of Lamb wave acoustic data. A coding method based on frequency-division multiplexing–pulse-position modulation (FDM-PPM) is proposed to address the low transmission rate in Lamb wave communication systems. Experimental results demonstrate that the proposed Lamb wave communication system can achieve a maximum transmission rate of up to 50 kbps with a bit error rate as low as 90.7%. Compared with methods using Amplitude-Shift Keying (ASK) and pulse-position modulation (PPM), this method effectively enhances the transmission rate of the Lamb wave communication system while reducing the energy consumption of the excitation signal.

## 1. Introduction

Traditional sensor networks primarily rely on wired data transmission. However, in certain specific scenarios, the structural complexity of the monitored components or harsh operating environments can limit the feasibility of wired transmission [1,2,3]. With their low cost and long-distance propagation advantages, Lamb waves have become an important technical means for structural condition monitoring [4,5,6]. In specific scenarios, complex working conditions limit the use of cables and electromagnetic waves. For instance, in the online monitoring systems of the aerospace field, the installation of cables compromises structural integrity and increases the difficulty of installation and maintenance. Although the use of electromagnetic wave wireless communication can achieve lightweight sensor networks, the Faraday effect causes metal panels to attenuate electromagnetic waves, significantly affecting the data transmission’s reliability. Additionally, the electromagnetic waves radiated outward by the sensor networks may interfere with onboard aircraft equipment, posing potential risks. Integrating Lamb waves from non-destructive testing as a carrier to achieve a sensing network that combines detection and communication is important for promoting the development of structural health monitoring networks [7].

Currently, Lamb wave data transmission’s modulation and demodulation methods include on-key control, phase-shift keying, frequency-division multiplexing, code-division multiple access, and other methods [8,9,10]. Kexel et al. took the sinusoidal pulse signal processed by the Hanning window as the carrier signal and modulated it using on–off keying, achieving a data transmission rate of 100 kbps on a glass fiber composite plate [11].

In addition, to address the Lamb wave communication among multiple sensor nodes, Zonzini et al. proposed a Lamb wave communication method based on time-reversal pulse position [12]. This method leverages the differences in transfer functions along different propagation paths to enable data transmission among multiple nodes on an aluminum plate. However, when the differences in transfer functions between sensor nodes are minor, these nodes cannot be effectively distinguished using the aforementioned method. The research team drew inspiration from code-division multiple access (CDMA) to address this. It employed Kasami sequences as spreading codes, which were modulated by single-cycle sinusoidal signals to generate Lamb wave CDMA signals [13]. Despite this, the CDMA method suffers from a low transmission rate and requires dispersion compensation for Lamb wave dispersion. To resolve these issues, Kexel et al. proposed a Lamb wave communication method based on FDM, which allocates different subcarrier frequencies in the frequency domain to enable data transmission among multiple nodes [14,15]. Building on this, Mälzer et al. divided the waveguide bandwidth in FDM sensor networks into communication and defect bands [16]. Subsequently, Zonzini et al. used finite element models and experiments to study the selection of Lamb wave excitation frequencies for FDM on stiffened plates, verifying that the proposed method achieves a high signal-to-noise ratio [17].

Bahouth et al. experimentally compared Lamb wave communication methods using on–off keying (OOK), ASK, and binary phase-shift keying (BPSK) and found that the BPSK method exhibited superior interference resistance [18,19,20]. Xu et al., starting from the perspective of sparse compression of Lamb waves, used a phase-sparse reconstruction method to recover information from Lamb wave data and proposed a pulse code frequency-division multiple access (PCF-DMA) method to achieve the multi-access transmission of Lamb wave data [21,22,23].

Due to the impact of Lamb wave multimodal and dispersive characteristics on communication signal encoding methods, many efficient communication techniques are challenging to apply to directly transmit Lamb wave acoustic data. The dispersive nature of Lamb waves causes signal waveform distortion and time delay and induces slight variations in subcarrier frequencies during propagation. Moreover, the power supply conditions for Lamb wave sensor nodes are usually stringent, and continuous communication can accelerate the energy consumption of the communication system [24,25]. Although existing Lamb wave communication methods have alleviated these issues to some extent, limitations such as low transmission rates and high energy consumption remain.

This paper proposes a Lamb wave-based encoding method using FDM-PPM to address these issues. By assigning carrier frequencies to sensor nodes and subsequently processing the Lamb wave acoustic data with FDM and PPM, this method achieves the efficient transmission of Lamb wave acoustic data in plate structures while significantly reducing the energy consumption of the communication system.

The remainder of this paper is organized as follows. Section 2 introduces the proposed Lamb wave-based FDM-PPM scheme and its procedure. Section 3 experimentally validates the FDM-PPM scheme’s bit error rate (BER) and communication rate. Section 4 discusses the characteristics of the proposed method and compares it with other traditional methods.

## 2. Lamb Wave Communication Method Based on FDM-PPM

### 2.1. Construction of FDM-PPM Signals

In designing the signals, this study thoroughly considered Lamb waves’ dispersion characteristics and multimode properties. Based on the frequency sweep curve of the piezoelectric patches in the experiments, the direct S_0_ mode Lamb wave signal was selected as the carrier for information transmission. In analyzing the signal duration, the direct S_0_ mode signal is ensured not to overlap with other mode signals in the time domain, thereby avoiding inter-symbol interference.

Within this limited time, rational encoding communication signals are the key to improving the communication rate. In sensor networks, the finite frequency bandwidth of the waveguide is typically divided and allocated to different sensor nodes. Each sensor node is assigned a specific set of carrier frequencies to distinguish between individual sensor nodes. This paper proposes an FDM-PPM modulation method for Lamb wave data transmission systems with several subcarriers. The core idea of this method is first to modulate the information to be transmitted using FDM and then perform secondary modulation on the phase and position of the signal pulse to obtain the information-carrying Lamb wave transmission signal.

#### 2.1.1. Phase Modulation of Frequency-Division Multiplexing Signals

Traditional PPM is a signal modulation technique with constant pulse width and amplitude, conveying information by controlling the position of the pulse within the symbol window [26]. In contrast, FDM modulates the transmitted information in the frequency domain using an on–off keying strategy, with each subcarrier mapping to a data bit. In this paper, based on PPM, FDM is applied to the signal pulse waveform to increase the number of information bits the signal can represent.

The FDM method modulates the transmission information in the frequency domain using an on–off keying (OOK) strategy, where each subcarrier maps to a single data bit. When the subcarrier frequency is excited, the transmitted data bit is a ‘1’, and otherwise, the transmitted data bit is a ‘0’. In this study, a Hanning window was used for excitation. The expression for a signal with N subcarriers is as follows:

(1) $$x_{FDM}(t)=0.5\times \left[1-\cos\left(\frac{2\pi }{T}\right)\right]\cdot \left[\sum_{i=1}^{N_{s}-1}\sin\left(2\pi f_{si}t+\phi_{si}\right)\right]$$

where *T* denotes the width of the windowing function, and *f_si_* denotes the frequency of the *i*-th subcarrier in the *s*-th subcarrier sequence; *Φ_si_ ∈ {0, π}* denotes the phase of the *i*-th signal in the *s*-th subcarrier sequence; and *x_FDM_* denotes the modulated signal.

In traditional FDM methods, to ensure that adjacent subcarriers do not interfere with each other, the frequency spacing between the center frequencies of subcarriers was set to be at least equal to the main lobe bandwidth of each subcarrier. To improve the waveguide frequency band utilization, the FDM-PPM method defines the two non-interfering subcarriers as the first sequence. It designates the subcarrier positioned between these two subcarriers as the second sequence. Only one of the two subcarriers is used when representing a single piece of information. Suppose the waveform of the first set of subcarriers is insufficient to represent the bit information adequately. In this case, the system switches to the second set of subcarriers for transmission, while the first set remains unexcited.

**Figure 1:**
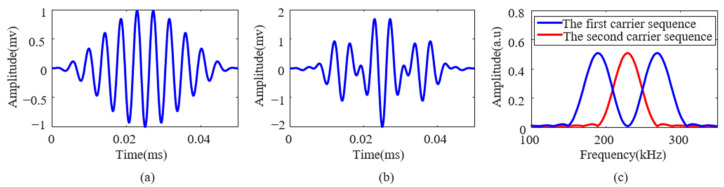
Illustrations of subcarrier signal modulation: (**a**) the first carrier sequence, (**b**) the second carrier sequence, and (**c**) the carrier spectrum.

A specific example for illustration: The first set of subcarriers consists of two subcarriers with frequencies of 190 kHz and 270 kHz, while the second set includes a subcarrier with a center frequency of 230 kHz. For a modulated signal representing the information “00101,” the signal waveform is shown in Figure 1a. However, when the modulated signal needs to represent “00110,” the first set of subcarriers has reached its capacity limit and cannot continue to carry subsequent bit information. At this point, the system switches from the first set of subcarriers to an unused state and activates the second set for modulation. Figure 1b shows the corresponding signal waveform, while Figure 1c displays the frequency-domain distribution of both sets of subcarrier signals.

#### 2.1.2. Position Modulation of Frequency-Division Multiplexing Signals

**Figure 2:**
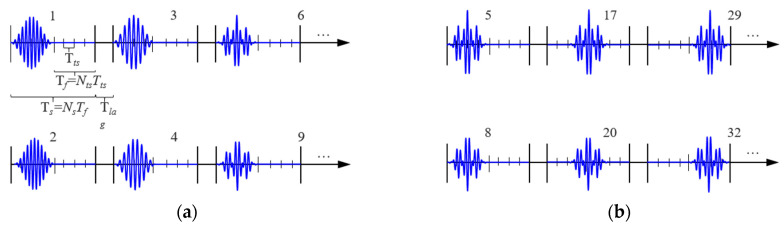
Schematic of FDM-PPM sequence structure: (**a**) signal waveform modulation and (**b**) signal position modulation.

Based on the subcarrier frequencies allocated to the sensor network nodes, the signal pulses are first modulated using FDM and then PPM, ultimately generating information-carrying FDM-PPM signals. Figure 2 illustrates the structure of the FDM-PPM sequence, where the numbers above the time axis correspond to the information content represented by different symbols. Parameter *T_la_* denotes the guard interval between each FDM-PPM signal, which is used to prevent mutual interference between signals. The time axis *T_s_* is divided into *N_s_* frames, each with a duration of *T_f_*. Each frame corresponds to a symbol, representing a signal modulated by frequency-division multiplexing.

A complete frame structure comprises *N_ts_* time slots, each with a duration of *T_ts_*. Within this structure, *T_ts_* not only represents the width of the time slot but also has a close relationship with the position of the signal pulse. Figure 2a illustrates the signal structure of FDM-PPM after only undergoing FDM processing. In contrast, Figure 2b presents the signal structure of FDM-PPM for the same pulse signal after modulation with different phases and positions. The expression for the FDM-PPM signal is

(2) $$x_{FDMPPM}=x_{FDM}*\sum_{k=0}^{\infty }\delta (t-lT_{ts})$$

where *x_FDM_* denotes the signal pulse modulated by PPM; * denotes the convolution operation; and l ∈ {0, 1, 2, …, *N_ts_*} denotes the number of time slots between the frame start position and the signal pulse. The number of bits represented by an FDM-PPM symbol, *I_FDMPPM_*, is

(3) $$I_{FDMPPM}=\log_{2}\left[2*\left(\sum_{k=0}^{1}2^{N-k}-1\right)*\left(N_{ts}+1\right)\right]$$

where *N* denotes the maximum number of subcarriers in a subcarrier sequence; and *k* denotes the number of adjacent subcarriers in a subcarrier sequence. The communication rate of the FDM-PPM system is

(4) $$R_{FDMPPM}=\frac{1}{T_{s}}\log_{2}\left[2*\left(\sum_{k=0}^{1}2^{N-k}-1\right)*\left(N_{ts}+1\right)\right]$$

**Figure 3:**
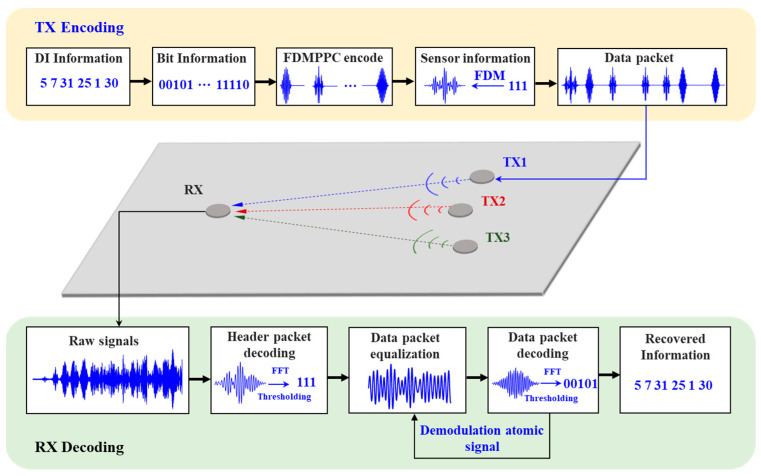
Schematic of Lamb wave-sensing network communication.

A flowchart of the Lamb wave FDM-PPM communication method is shown in Figure 3.

### 2.2. Design of FDM-PPM Signal Structure

**Figure 4:**
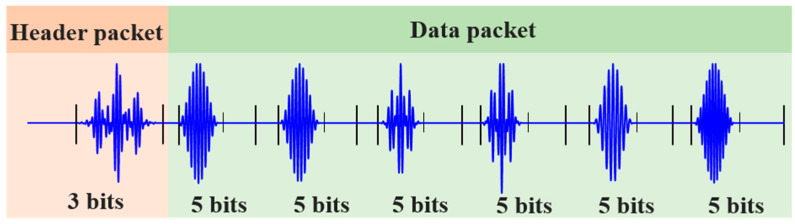
The structure of packets used in the Lamb wave communication protocol.

In the FDM-PPM communication system, a data set consists of one header packet and multiple data packets. For example, a structure containing one header packet and six data packets is illustrated in Figure 4. The header packet carries the identification information of the transmitting sensor in the sensor network, with a signal time width of *T_s_*. Moreover, the header packet not only marks the beginning of the group of data packets but also assists in locating the positions of the subsequent data packet signals. Its signal waveform is obtained through FDM modulation. In the FDM-PPM communication system, each data packet contains multiple FDM-PPM signals, which carry information regarding the path damage between the upper-level sensing nodes monitored with Lamb waves. The time width of each frame signal within the FDM-PPM is *T_f_*, while the total time width of the entire signal is *T_s_*.

**Figure 5:**
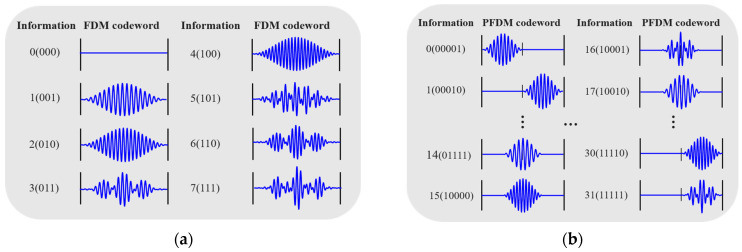
Data packet-encoding book: (**a**) header-packet-encoding book and (**b**) data-packet-encoding book.

Figure 5 illustrates the designed signal encoding sets for the header packet and data packets. The signal for the header packet is obtained through the FDM method, featuring eight distinct signal waveforms, each capable of carrying three bits of information. In contrast, the signals for the data packets are obtained via FDM-PPM, comprising 32 different signal waveforms, each capable of carrying five bits of information.

### 2.3. The FDM-PPM Decoding Method

**Figure 6:**
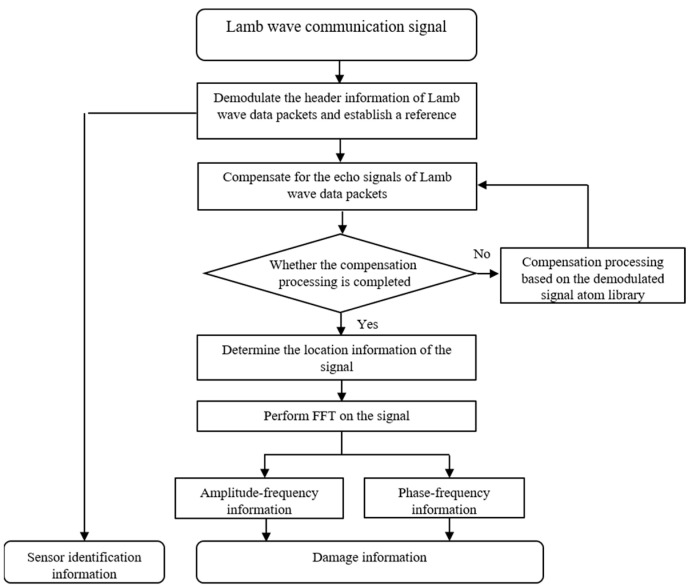
Schematic of subcarrier signal modulation.

Information is transmitted as data packets in the inter-node communication protocol based on the Lamb wave communication sensor network described above. After receiving the FDM-PPM signal, the sensor node first performs equalization on the Lamb wave echo signal to compensate for distortion and attenuation during signal transmission. Subsequently, the node conducts demodulation operations to recover the initially transmitted information from the modulated signal. The decoding workflow of the FDM-PPM signal is illustrated in Figure 6, and its detailed process can be summarized as follows:

Step 1: Pre-capture signals of different combinations of header packets and data packets under single excitation to construct a demodulation atom library.

Step 2: Demodulate the signal of the header packet and identify the key position within the signal, which serves as the reference for the demodulation of subsequent data packet signals.

Step 3: Along the time axis, extract the next data packet signal, perform equalization on it, and determine its central position. This position serves as the reference for subsequent data packet echo signals.

Step 4: Demodulate the signal pulse positions within the data packet to extract the pulse signals’ location information.

Step 5: Carry out a Fourier transform on the signal pulses within the data packet to obtain their amplitude–frequency and phase–frequency characteristics.

Step 6: Complete the demodulation of the transmitted information based on the location, amplitude–frequency, and phase–frequency information of the FDM-PPM signals in conjunction with the corresponding code table.

Step 7: Repeat Steps 3 to 6 until all information transmitted by the FDM-PPM communication system is demodulated.

### 2.4. Experimental Setup

**Figure 7:**
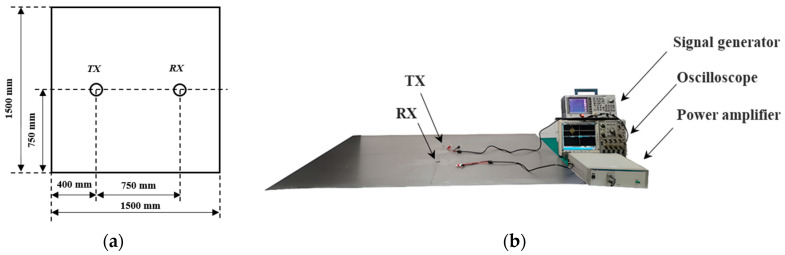
(**a**) Layout of sensor node in the experiment and (**b**) experimental platform.

The Lamb wave FDM-PPM acoustic communication experimental platform is illustrated in Figure 7. The experiment was conducted on a Q235 steel plate measuring 1.5 m in length and 1.8 mm in thickness. The material properties of the steel plate include Young’s modulus of 210 GPa, a Poisson’s ratio of 0.3, and a density of 7890 kg/m^3^. Two circular ceramic piezoelectric patches, made of PZT-5 material, were affixed to the front side of the steel plate, designated for the excitation and reception of Lamb wave signals, respectively. The patches have a diameter of 10 mm and a thickness of 1 mm. A signal generator (Tektronix, Beaverton, OR, USA) and a single-channel power amplifier (TEGAM, Cleveland, OH, USA) were employed to drive the piezoelectric disks. A four-channel oscilloscope (Tektronix, Beaverton, OR, USA) with a maximum sampling rate of 2 GHz was used for signal acquisition at the receiving end.

## 3. Analysis of the Experimental Results for Lamb Wave FDM-PPM

### 3.1. Lamb Wave Continuous Data Transmission Experiment Based on FDM-PPM

**Figure 8:**
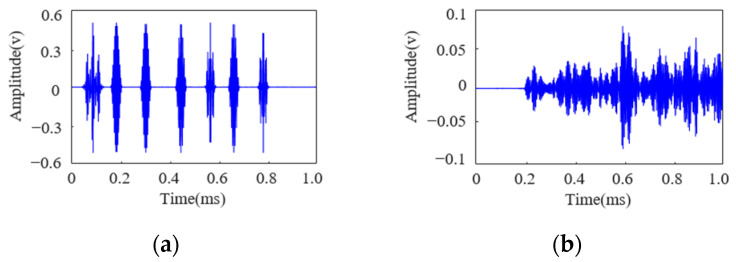
(**a**) FDM-PPM excitation signal and (**b**) FDM-PPM received signal.

Based on the layout of the sensor nodes and the available bandwidth, the signal parameters were designed in this study as follows: the signal duration was set to 0.1 ms, the frame width *T_f_* was 50 μs, the number of time slots *N_ts_* was 4, and the combination of subcarrier frequencies at 190 kHz, 230 kHz, and 270 kHz was selected. On this basis, Lamb waves were employed to transmit signals with varying numbers of symbols (ranging from two to seven), with each symbol encoding five bits of information. For example, a signal comprising seven symbols is presented (encoded information: 111 00111 00011 10001 10010 01000 10010), and the corresponding excitation signal and received Lamb wave signal are illustrated in Figure 8.

**Figure 9:**
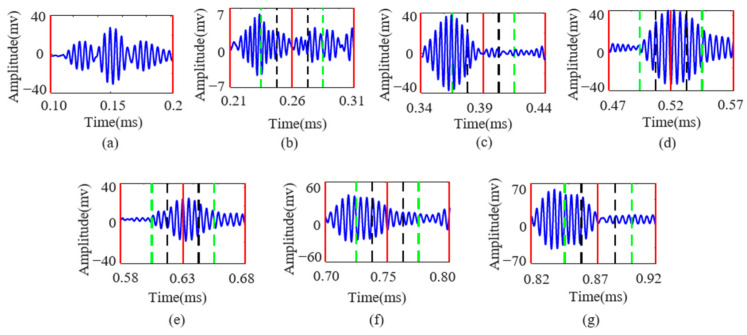
(**a**–**g**) intercepted Lamb wave FDM-PPM signals corresponding to excitation numbers 1 to 7, respectively.

As observed in Figure 8, the influence of the structural boundaries causes significant overlap between the direct wave and the first few reflected waves of the Lamb wave, rendering the demodulation of transmitted information using matched filtering methods impractical. Moreover, with seven distinct signal waveforms, employing cross-correlation methods would require simultaneous cross-correlation operations on multiple signals, significantly increasing the computational load for demodulation. According to the method illustrated in Figure 6, constructing a demodulation atom library allows for compensation of the Lamb wave echo signals. The compensated signals are shown in Figure 9.

Figure 9 illustrates the Lamb wave signal after equalization compensation containing seven symbols. In the figure, the red lines mark the starting position, the 1/2 position, and the ending position of the signal; the green lines correspond to the 1/4 and 3/4 positions of the signal; and the black lines indicate the 3/8 and 5/8 positions. In benchmarking the signal wave packets, the location information carried by the signal can be precisely obtained. Additionally, the frequency and phase information of the signal is extracted through the Fourier transform, thereby enabling the complete recovery of the transmitted information.

To verify the effectiveness of the proposed equalization algorithm in demodulating FDM-PPM signals, this study conducted multiple transmission and demodulation experiments on Lamb wave FDM-PPM signals with varying numbers of symbols (ranging from two to seven). The results demonstrate that the algorithm can effectively address the demodulation requirements for signals with different symbol counts. The detailed demodulation performances are presented in Table 1.

As observed in Table 1, when employing the Lamb wave FDM-PPM method for data transmission, the demodulation accuracy decreases gradually with an increased number of symbols. In this Lamb wave communication system, the maximum communication rate achievable using the FDM-PPM method is 50 kbps. During the experiment, 970 bits of information were transmitted, of which 880 bits were successfully demodulated, yielding a system demodulation accuracy of 90.7%.

### 3.2. Analysis and Discussion of Bit Error Rate

**Figure 10:**
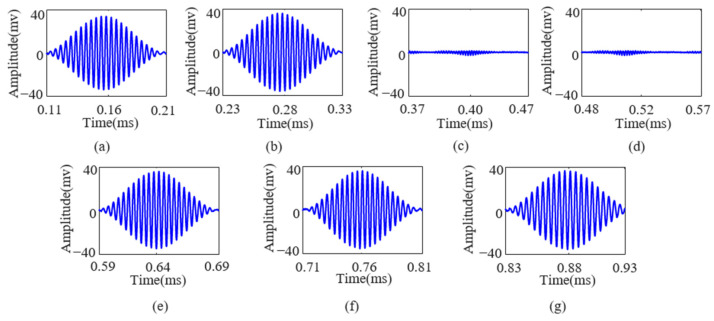
(**a**–**g**) Intercepted Lamb wave ASK signals corresponding to excitation numbers 1 to 7, respectively.

**Figure 11:**
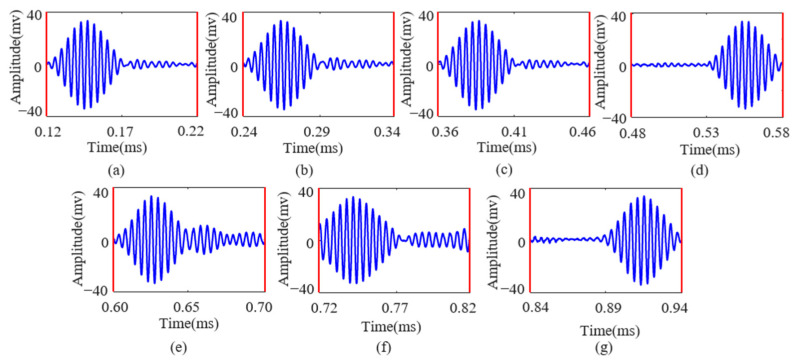
(**a**–**g**) Intercepted Lamb wave PPM signals corresponding to excitation numbers 1 to 7, respectively.

To demonstrate the superiority of the FDM-PPM method over other modulation techniques, this study conducted comparative tests on ASK and PPM signals with varying numbers of symbols (ranging from two to seven). The following discussion focuses on the example of ASK and PPM signals with seven symbols. The demodulation results of the ASK and PPM signals after compensation for Lamb wave echoes are shown in Figure 10 and Figure 11, respectively.

Figure 10 and Figure 11 show that the Lamb wave echo equalization algorithm effectively compensates for the echoes of continuously transmitted ASK and PPM signals. In Figure 11, the red line indicates the start and end positions of the PPM signal, respectively. After processing with this algorithm, the information transmitted by the ASK and PPM signals can be accurately demodulated. This study conducted multiple transmission and equalization experiments on ASK and PPM signals with varying numbers of symbols (ranging from two to seven). The results indicate that the demodulation performance of ASK and PPM signals is satisfactory after equalization. The detailed results are presented in Table 2 and Table 3, respectively.

Due to the different modulation principles of the ASK and PPM methods, the number of transmitted bits also varies. Specifically, the ASK method transmitted 421 bits of information, all correctly demodulated after equalization, achieving a demodulation accuracy of 100%. In contrast, the PPM method transmitted 512 bits of information, of which 498 bits were correctly demodulated, resulting in a demodulation accuracy of 97.2%. Following the experiment on single-frequency communication signals of Lamb waves, subsequent experiments were conducted on the continuous transmission of Lamb waves using FDM. The detailed experimental results are presented in Table 4.

In summary, when continuously transmitting Lamb wave ASK data on the constructed plate, the Lamb wave echo equalization compensation algorithm is used to demodulate the data, ensuring correct demodulation with a system communication rate of 10 kbps. Using the PPM-based method for Lamb wave data transmission achieves an accuracy of 97.26%, with a system communication rate of 20 kbps. The FDM-based method for Lamb wave data transmission achieves an accuracy of 98.58%, with a system communication rate of 30 kbps. Employing the FDM-PPM modulation method for Lamb wave data transmission achieves an accuracy of 90.7%, with a system communication rate of 50 kbps. The experimental results indicate that the ASK method has the lowest bit error rate but a relatively lower transmission rate. In contrast, the FDM-PPM method has the highest but relatively higher bit error rate.

The experimental results show that the dispersion characteristics of Lamb waves cause distortion in the signal waveform, thereby significantly affecting the signal’s phase discrimination. The ASK method takes the complete waveform of the direct S_0_ mode Lamb wave signal as the analysis object and does not need to consider the phase information of the signal. Therefore, the signal waveform distortion caused by Lamb wave dispersion and equalization compensation errors will not significantly affect the demodulation results. However, PPM and FDM-PPM signals carry phase information, and the FDM-PPM signal has a broader frequency band, further complicating the signal’s phase discrimination. To address the high error rates caused by Lamb wave dispersion, channel coding techniques such as Hamming and convolutional codes can enhance the error correction capability of the transmitted information. The FDM-PPM method proposed in this study slightly reduces demodulation accuracy compared with the ASK, PPM, and FDM methods. However, it achieves a significant increase in transmission rate, with the excitation signal’s energy being only half that of the other methods.

## 4. Discussion

### 4.1. Parameter Analysis of FDM-PPM

**Figure 12:**
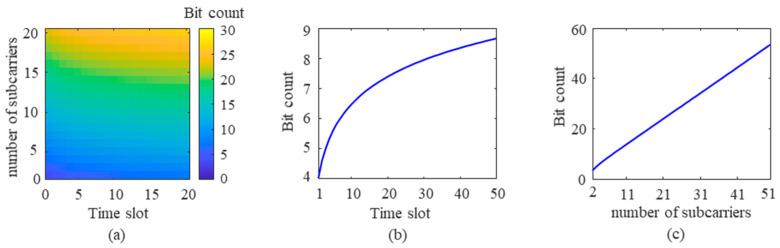
The impact of FDM-PPM signal parameters on bit number: (**a**) the number of time slots, (**b**) the number of time slots, and (**c**) the number of subcarriers.

According to Equation (3), the maximum number of bits an FDM-PPM signal can represent is closely related to the length of the time slots and the number of subcarriers. When the number of subcarriers is held constant, increasing the number of time slots in the signal raises the maximum number of bits the signal can represent. Conversely, if the number of time slots remains unchanged, increasing the number of subcarriers can also enhance the maximum number of bits the signal can represent. The following discussion will delve into the specific impact of FDM-PPM signal parameters on improving the communication rate based on the modulation principle of the proposed method. Figure 12a illustrates the trend of how changes in FDM-PPM signal parameters affect the number of bits.

Figure 12a demonstrates a close relationship between the number of bits in the signal and the number of time slots and subcarriers. Using a specific example, the following discussion explores the trend of another parameter’s influence on the number of bits when the numbers of subcarriers and time slots are fixed. Figure 12b shows the trend of how the number of time slots affects the number of representable bits when the number of subcarriers is fixed at 2. It is evident from the figure that the increase in the number of bits is more pronounced when the number of time slots is low. However, as the number of time slots increases, the growth rate of the number of bits gradually slows down. This is because the interval between signal pulses narrows with an increase in time slots, which may adversely affect the accurate demodulation of the transmitted information. Figure 12c illustrates the trend of how the number of subcarriers affects the number of representable bits when the number of time slots is fixed at 4. The figure shows that the number of bits increases approximately linearly with the number of subcarriers.

### 4.2. Comparison of the FDM-PPM with ASK and PPM Method

**Figure 13:**
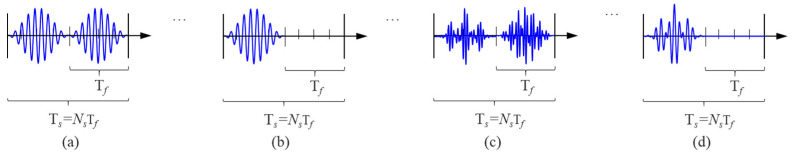
Schematics of the duration of different types of symbol signals: (**a**) ASK, (**b**) PPM, (**c**) FDM, and (**d**) FDM-PPM.

Based on the modulation principle of the FDM-PPM method, it significantly enhances the communication rate of the system compared with ASK, PPM, and FDM. The following section will explore the relationship between the number of signal bits, communication rate, and excitation signal energy with the time slot length and compare the performance differences between FDM-PPM and the other modulation methods. Specifically, the ASK method is independent of the number of time slots and subcarriers; the FDM method is related to the number of time slots but not to the number of subcarriers, whereas the PPM method is related to the number of subcarriers but independent of the number of time slots. In contrast, the longer the signal duration of the FDM-PPM method, the more bits can be transmitted within the same period. A schematic comparison of the symbol signal duration between the FDM-PPM method and the aforementioned three methods is shown in Figure 13.

**Figure 14:**
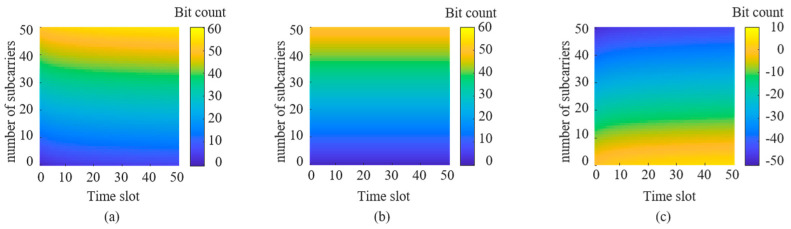
Comparison of bit number differences between FDM-PPM signals and other modulation methods when *N_s_* = 2: (**a**) ASK, (**b**) PPM, and (**c**) FDM.

As illustrated in Figure 1 and Figure 13, the signal duration for ASK and FDM is *T_f_*, whereas the signal duration for PPM and FDM-PPM is *T_s_*, where *T_s_* equals Ns multiplied by *T_f_ (T_s_ = N_s_ × T_f_)*. Consequently, multiple ASK and FDM signals can be accommodated within the duration of one FDM-PPM signal. Under the same signal duration condition, there are significant differences in signal bits and communication rates among ASK, FDM, PPM, and FDM-PPM. Figure 14 shows the specific differences in the number of bits between FDM-PPM and the aforementioned three methods when *N_s_* equals 2.

**Figure 15:**
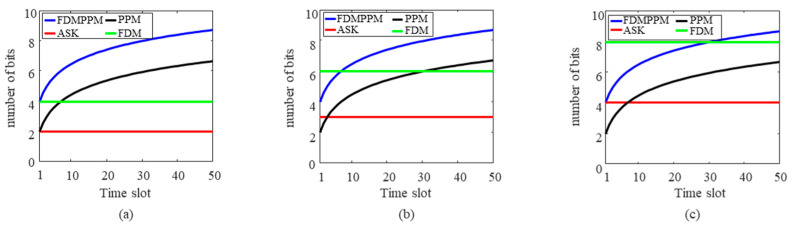
Bit number differences in symbol signals with different frame numbers when the number of subcarriers is 2: (**a**) ASK, (**b**) PPM, and (**c**) FDM.

Figure 14 demonstrates that the number of bits transmitted using the FDM PPM method significantly increases compared to the ASK and PPM methods. When the number of subcarriers is low, the FDM-PPM method exhibits a higher bit count compared with FDM; however, as the number of subcarriers grows, the bit count of FDM signal transmission gradually surpasses that of FDM-PPM. According to Equation (2), the energy of the FDM-PPM signal is only 1/*N_s_* the energy of ASK and FDM signals (where *N_s_* is the number of frames) and is comparable to the energy of PPM signals. Figure 15 illustrates the differences in the number of bits for various frame numbers when the number of subcarriers is 2.

Figure 15 reveals the bit performance of FDM-PPM signals under different frame numbers. When *N_s_* is 2, the number of bits in the FDM-PPM signal is significantly higher than that of the other three modulation methods. However, when *N_s_* increases to 3, although the number of bits in the FDM-PPM signal is higher than that of ASK and PPM, it only exceeds that of FDM when the number of time slots is greater than 7. Furthermore, when *N_s_* is 4, the number of bits in the FDM-PPM signal only surpasses that of FDM when the number of time slots exceeds 31. These results indicate that the bit advantage of FDM-PPM signals gradually emerges with increasing frame and time slot numbers, but their dependence on the number of time slots is also more pronounced.

Based on the aforementioned analysis, FDM-PPM signals, when employing a small number of subcarriers, not only transmit a more significant number of bits but also exhibit the advantage of lower transmission-signal energy compared with ASK, PPM, and FDM signals. As the number of subcarriers increases, the bit count of FDM-PPM signals surpasses that of ASK and FDM signals. Although the bit count of FDM-PPM remains lower than that of FDM, its signal energy is relatively lower. Moreover, an increase in subcarriers generally leads to a decline in transmission accuracy across various communication methods. In sensor networks with limited bandwidth that require subcarrier allocation for multiple nodes, the method proposed in this study demonstrates significant advantages over the other three methods, making it particularly suitable for communication between sensor network nodes with constrained power consumption.

## 5. Conclusions

This study proposes a Lamb wave-based FDM-PPM data transmission scheme in plate structures, leveraging the unique dispersive characteristics of Lamb waves. Compared with ASK, PPM, and FDM, the proposed FDM-PPM method effectively enhances the system’s communication rate. It reduces the signal energy consumption at the excitation end. Experimental results demonstrate that the Lamb wave communication based on FDM-PPM can achieve a maximum rate of up to 50 kbps with a bit error rate as low as 90.7%. Moreover, when the condition of the monitored component changes, the scheme can adapt to the new channel conditions and continue data transmission by reacquiring Lamb wave symbol signals with different carrier combinations and updating the demodulation atom library. The proposed method effectively improves the transmission rate of Lamb wave communication systems, providing new guidance for low-power Lamb wave sensing network communication. Additionally, this scheme is universal, applicable to Lamb wave communication, and easily extendable to electromagnetic wave communication, showing great potential for application.

Further research will focus on addressing communication issues when structural defects occur, aiming to develop more robust adaptive equalization compensation algorithms. These algorithms will dynamically adapt to channel variations and optimize signal transmission quality. Simultaneously, further research will be conducted on joint control strategies for multi-sensor node communication using Lamb waves to achieve efficient multi-node communication. Additionally, integrating structural defects and damage identification results in a Lamb wave-sensing network that combines defect detection and data transmission capabilities, comprehensively improving the system’s overall performance and application value.

## Figures and Tables

**Table 1:** The demodulation results of the Lamb wave FDM-PPM signals.

Number of Symbols	Number of Transmitted Bits	Number of Correctly Demodulated Bits	Demodulation Accuracy
2	60	60	100%
3	90	70	77%
4	140	140	100%
5	160	140	87.5%
6	240	220	91.6%
7	280	250	89.3%

**Table 2:** The demodulation results of the Lamb wave ASK signals.

Number of Symbols	Number of Transmitted Bits	Number of Correctly Demodulated Bits	Demodulation Accuracy
2	12	12	100%
3	18	18	100%
4	36	36	100%
5	75	75	100%
6	132	132	100%
7	148	148	100%

**Table 3:** The demodulation results of the Lamb wave PPM signals.

Number of Symbols	Number of Transmitted Bits	Number of Correctly Demodulated Bits	Demodulation Accuracy
2	36	36	100%
3	72	72	100%
4	80	80	100%
5	90	90	100%
6	108	101	93.5%
7	126	119	94.4%

**Table 4:** The demodulation result of the Lamb wave FDM signals.

Number of Symbols	Number of Transmitted Bits	Number of Correctly Demodulated Bits	Demodulation Accuracy
2	60	60	100%
3	90	70	77%
4	140	140	100%
5	160	140	87.5%
6	240	220	91.6%
7	280	250	89.3%

## Data Availability

The data presented in this study are available on request from the corresponding author.
